# Characterization, bio-uptake and toxicity of polymer-coated silver nanoparticles and their interaction with human peripheral blood mononuclear cells

**DOI:** 10.3762/bjnano.12.23

**Published:** 2021-03-24

**Authors:** Sahar Pourhoseini, Reilly T Enos, Angela E Murphy, Bo Cai, Jamie R Lead

**Affiliations:** 1 Department of Ophthalmology, Baylor College of Medicine, Houston, Texas, 77030, United States; 2 Department of Pathology, Microbiology, and Immunology, School of Medicine, University of South Carolina, Columbia, SC, 29209, United States; 3 Department of Epidemiology and Biostatistics, Arnold School of Public Health, University of South Carolina, Columbia, SC, 29208, United States; 4 Center for Environmental Nanoscience and Risk, Department of Environmental Health Sciences, Arnold School of Public Health, University of South Carolina, Columbia, SC, 29208, United States

**Keywords:** human peripheral blood mononuclear cells, silver ions, silver nanoparticles, toxicity, uptake

## Abstract

Silver nanoparticles (AgNPs) are widely used in medical applications due to their antibacterial and antiviral properties. Despite the extensive study of AgNPs, their toxicity and their effect on human health is poorly understood, as a result of issues such as poor control of NP properties and lack of proper characterization. The aim of this study was to investigate the combined characterization, bio-uptake, and toxicity of well-characterized polyvinylpyrrolidone (PVP)-coated AgNPs in exposure media during exposure time using primary human cells (peripheral blood mononuclear cells (PBMCs)). AgNPs were synthesized in-house and characterized using a multimethod approach. Results indicated the transformation of NPs in RPMI medium with a change in size and polydispersity over 24 h of exposure due to dissolution and reprecipitation. No aggregation of NPs was observed in the RPMI medium over the exposure time (24 h). A dose-dependent relationship between PBMC uptake and Ag concentration was detected for both AgNP and AgNO_3_ treatment. There was approximately a two-fold increase in cellular Ag uptake in the AgNO_3_ vs the NP treatment. Cytotoxicity, using LDH and MTS assays and based on exposure concentrations was not significantly different when comparing NPs and Ag ions. Based on differential uptake, AgNPs were more toxic after normalizing toxicity to the amount of cellular Ag uptake. Our data highlights the importance of correct synthesis, characterization, and study of transformations to obtain a better understanding of NP uptake and toxicity. Statistical analysis indicated that there might be an individual variability in response to NPs, although more research is required.

## Introduction

Nanotechnology is a rapidly-growing industry that creates nanoscale products with novel physicochemical properties [1]. Nanoparticles (NPs) are particles with at least one dimension between 1 and 100 nm, which gives them unique properties for innovative applications [2]. NPs are present in numerous commercial products such as cosmetics, electronics, and textiles. Also, they are widely used in industry, including various biomedical and drug-delivery applications for the treatment of diseases [3–6]. Silver nanoparticles (AgNPs) are one of the most frequently used NPs in commercial products in health and medicine [7–9] due to their antibacterial and antiviral properties [10–11]. In fact, the use of AgNPs in commercially available products is anticipated to double in the next five years [12]. AgNPs have the potential to prevent bacterial colonization on different surfaces, such as catheters and prostheses [13–14], which raises concern about Ag release in the body and their potential negative impact on human health. For instance, the silver concentration in the blood of people without any occupational or medicinal exposure is less than 1 µg·L^−1^ [15]. However, in burn patients who are treated with silver containing antibacterial creams, Ag in the blood can be found up to a concentration of 310 µg·L^−1^ [15]. Additionally, one of the main routes of exposure in humans is reported as oral exposure since AgNPs are used in the food industry (in food packaging or in food processing machines as coating). They found that Ag in doses relevant for human intake might alter gut microbiota and modulate the systemic homeostasis of the intestinal tract [16–17].

A human body might be exposed to AgNPs through injection, ingestion, inhalation, as well as dermal and ocular contact [18]. As AgNPs enter the body through injection or degradation of biomaterials, they are translocated to the circulatory system and can come in direct contact with human peripheral blood mononuclear cells (PBMCs) before ultimately being distributed to the main organs where they can accumulate [19]. PBMCs are primary immune cells and mainly consist of monocytes and lymphocytes (B and T cells) and are responsible for the cellular host defense against foreign substances [20]. These cells secrete a variety of mediators depending on the environmental stimuli, including pro- and anti-inflammatory cytokines. Despite the increased usage of and the likely increased exposure to AgNPs, there is a lack of quantitative analysis of bio-uptake and potential cytotoxicity in PBMCs after exposure to well-characterized AgNPs. A number of studies have investigated the bioavailability and cytotoxicity of AgNPs using different cell lines and rodent models [7,21–23]. However, some of these cell lines are resistant to toxicity effects that may be induced by AgNPs [24]. Furthermore, there are only a few studies on the bio-uptake and exposure of AgNPs in human PBMCs [25–34]. Few, if any, of these studies fully discuss procurement/synthesis, characterization, transformations in exposure media, and bio-uptake and toxicity of NPs at relevant exposure concentrations with appropriate controls.

In this study we have used tightly constrained AgNPs, which have been well characterized, including their transformations during exposure. Additionally, we have assessed bio-uptake quantification, using mass spectrometry, and toxicity/cell viability after exposing human PBMCs to AgNPs at clinically relevant concentrations. We also compared the toxicity of AgNPs with dissolved Ag in PBMCs to distinguish between the effects of metallic Ag and metal ions. In addition, we investigated if there was significant variability across different individuals during various comparisons.

## Results

### PVP-AgNP characterization

In this study we used a multimethod approach to characterize pristine AgNPs, including dynamic light scattering (DLS), UV–vis spectroscopy (UV–vis), transmission electron microscopy (TEM), and inductively coupled plasma mass spectrometry (ICP-MS). DLS gave a *Z*-average hydrodynamic size of 33.7 ± 0.7 nm (mean ± standard deviation) and a PDI (polydispersity index) of 0.18, indicating an acceptably monodisperse particle suspension and an absence of larger agglomerates in ultrahigh-purity water (UHPW) (Table 1). A representative curve is shown in Supporting Information File 1, Figure S1a. The zeta potential was measured as −23.3 ± 2.1 mV for PVP-coated AgNPs (PVP-AgNPs). UV–vis spectra exhibited a characteristic absorption peak at 390 nm for Ag (Supporting Information File 1, Figure S1b). TEM imaging of the AgNP stock indicated a mean core size of 16.9 ± 0.3 nm and confirmed a spherical shape of NPs (Supporting Information File 1, Figure S1c and Figure S1d), which were shown to be silver by using EDX analysis (Supporting Information File 1, Figure S1e). Peaks of other elements (C, Cu, and O) in EDX analysis were observed due to grid material that was used for sample preparation. DLS estimates the hydrodynamic size of NPs, which includes the polymer and hydration shell around NPs and should be the same as, or larger than, the core size of NPs measured by TEM. Characterization values for pristine PVP-AgNPs can be found in Table 1.

**Table 1:** NP characterization results. Characterization of PVP-AgNPs in UHPW. DLS and TEM sizes are reported as mean ± standard deviation.

Parameter	Measurement

DLS size (nm)	33.7 ± 0.7
polydispersity index (PDI)	0.18
position of maximum UV–vis absorbance (nm)	390
TEM size (nm)	16.9 ± 0.3

**Figure 1:**
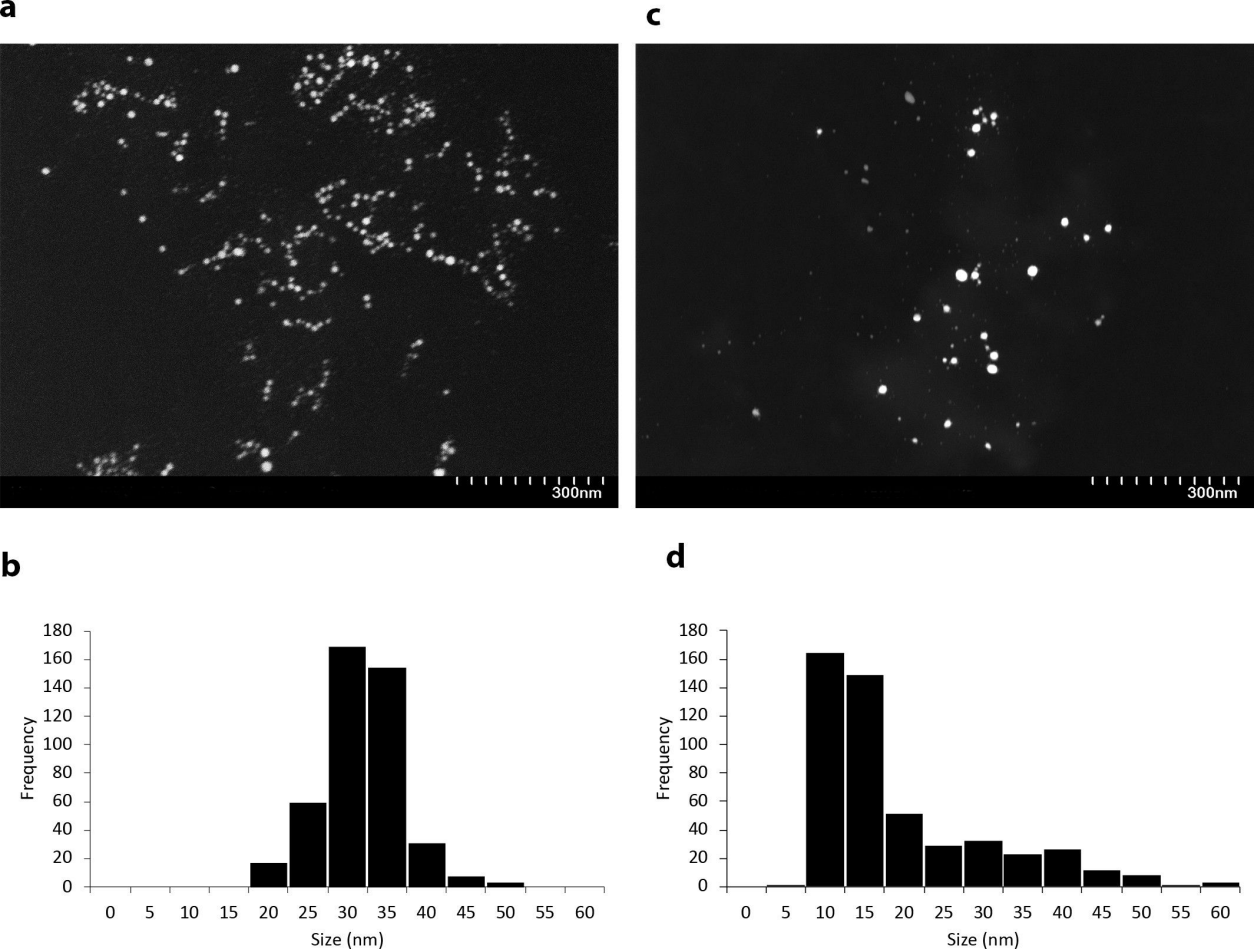
STEM images of 1000 µg·L^−1^ PVP-AgNPs and particle size distribution obtained from ImageJ. PVP-AgNPs in RPMI medium (without cells) at (a, b) *t* = 0, size distribution = 29 ± 0.49 nm and at (c, d) *t* = 24 h, size distribution = 17 ± 11 nm, under the same conditions as during exposure to cells (incubator at 37 °C with 5% CO_2_).

**Figure 2:**
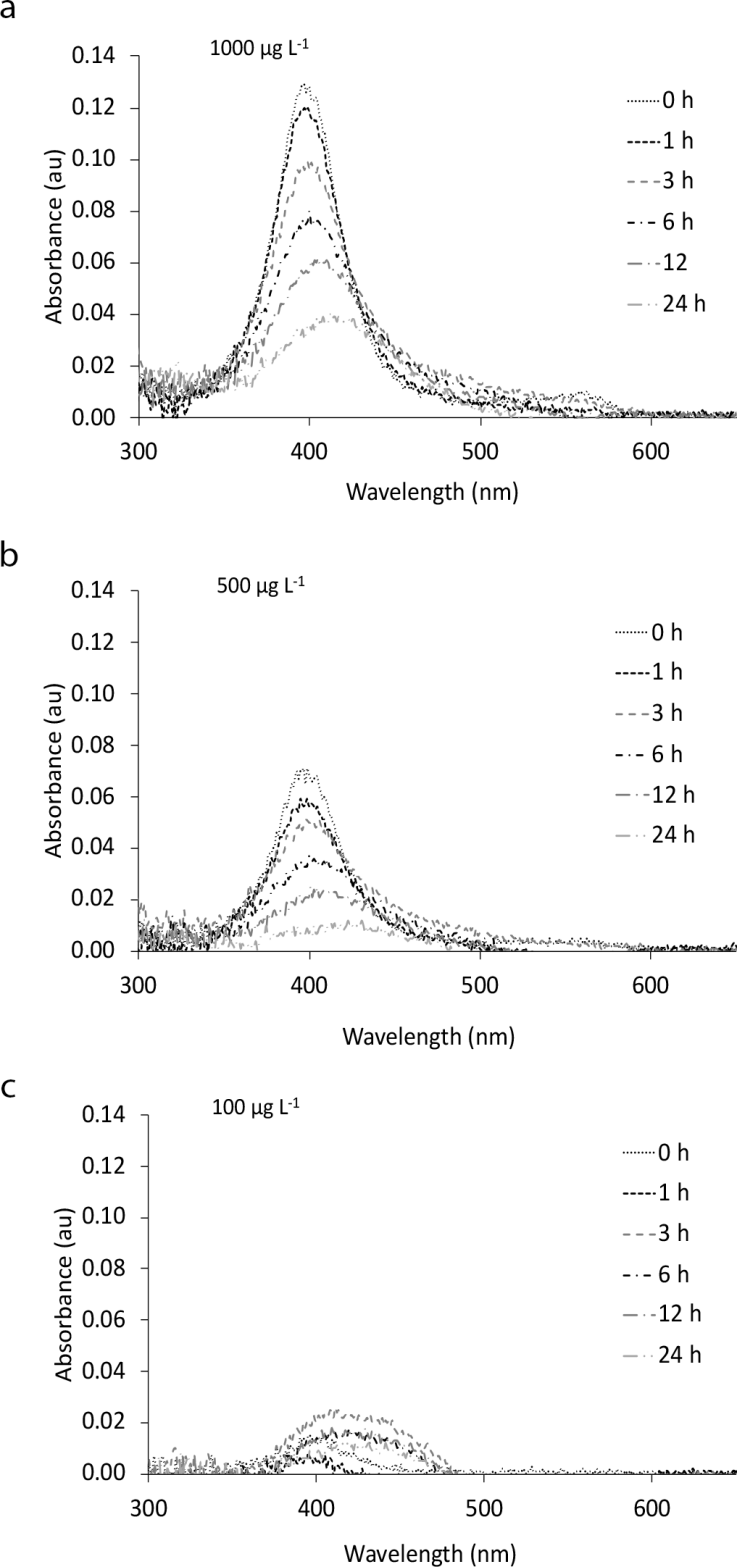
Behavior of PVP-AgNPs in RPMI medium during 24 h of exposure. UV–vis spectra of PVP-AgNPs in RPMI medium at concentrations of (a) 1000, (b) 500, and (c) 100 µg·L^−1^ as a function of the time. The lowest concentration (10 µg·L^−1^ PVP-AgNP) did not show any signal due to the detection limit of the UV–vis measurement.

Scanning transmission electron microscopy (STEM) images of 1000 µg·L^−1^ PVP-AgNPs in RPMI medium at the beginning of the experiment (*t* = 0) and after 24 h of exposure (*t* = 24 h) are shown in Figure 1. STEM images showed a mean size of 29 ± 4.9 nm at *t* = 0 in RPMI (Figure 1a), which is larger than the size of pristine NPs in UHPW. After 24 h, the PVP-AgNPs remained spherical, with a mean size of 17 ± 11 nm (Figure 1c). The images do not show any evidence of aggregation of NPs. Figure 1b and Figure 1d show a skewed distribution and a greater polydispersity of PVP-AgNPs in RPMI medium after 24 h of exposure. As a comparison, the extinction spectra of PVP-AgNPs at concentrations of 100, 500 and 1000 µg·L^−1^ in RPMI medium after 24 h was measured and the results are presented in Figure 2. A decrease in the UV–vis absorbance was observed for all concentrations. A small redshift and a broadening of the Ag peak, especially for the 1000 µg·L^−1^ PVP-AgNP concentration was observed after 24 h of exposure. The values of full width at half maximum (FWHM) for both 500 and 1000 µg·L^−1^ concentration increased (44 to 81 nm and 49 to 140 nm for 500 and 1000 µg·L^−1^, respectively) after 24 h of exposure. Supporting Information File 1, Table S1 shows extinction coefficients calculated according to the Beer–Lambert law, as described elsewhere [35].

### Measurement of dissolved Ag using ICP-MS

**Figure 3:**
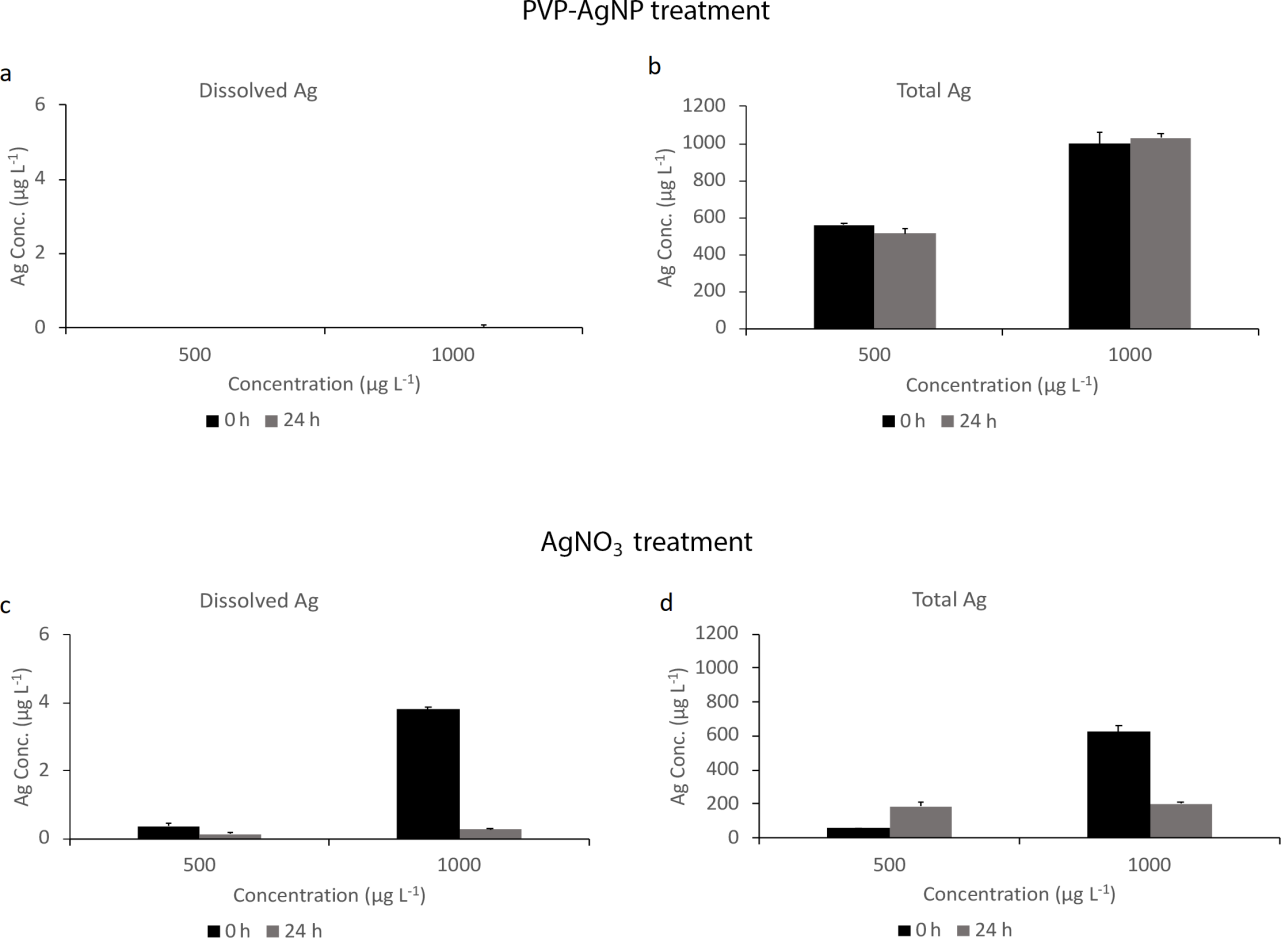
Dissolved and total Ag concentration in PVP-AgNPs and AgNO_3_. Dissolved Ag concentration measurements using centrifugal ultrafiltration units (a and c), and total Ag concentration (b and d) in RPMI medium at *t* = 0 and *t* = 24 h measured by ICP-MS. All experiments were performed in triplicates and are reported as mean ± SE (*n* = 3).

A solution of Ag in the culture medium (RPMI without added cells) was measured after addition of PVP-AgNPs at *t* = 0 (sample collection was performed immediately after addition of AgNP) and *t* = 24 h. For PVP-coated AgNPs, the concentration of Ag was below the limit of detection of the ICP-MS measurement (Figure 3a). The total Ag concentrations, however, showed no significant change (Figure 3b). A similar experiment for AgNO_3_ is shown in Figure 3c and Figure 3d, which shows 0.4% of Ag in the dissolved fraction and 60% in the total Ag fraction for a concentration of 1000 µg·L^−1^, with the remainder lost to surfaces such as containers or filter membranes. To confirm the results in the medium, and to compare the dissolution in RPMI and UHPW, the dissolution test was repeated using AgNO_3_ in UHPW and RPMI medium at two concentrations (500 and 1000 µg·L^−1^). Results indicate a near total recovery of total Ag in UHPW at both concentrations and Ag losses in RPMI medium and in ultrafiltered samples. A representative figure for the measurement of dissolved and total Ag for AgNO_3_ in UHPW and RPMI is shown in Supporting Information File 1, Figure S2.

### Ag uptake to the cells and mass balance

**Figure 4:**
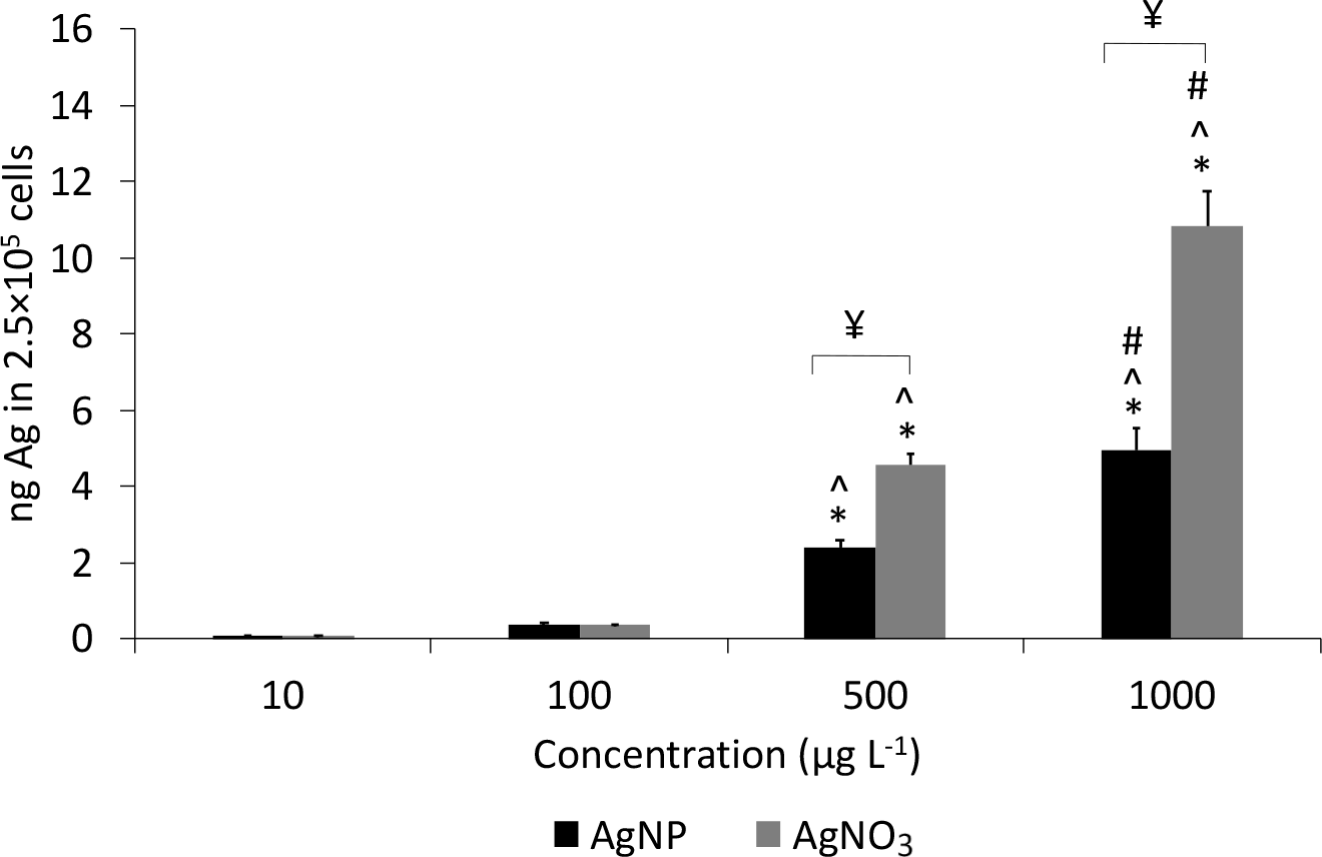
Ag uptake (ng) in 2.5 × 10^5^ cells. ICP-MS measurements of Ag that was adsorbed or attached to the cell surface after treatments with PVP-AgNPs and AgNO_3_ after 24 h of exposure. Data are presented as mean ± SE (*n* = 6). * statistically different from 10 µg·L^−1^, ^ statistically different from 100 µg·L^−1^, # statistically different from 500 µg·L^−1^, and ¥ statistically different when compared with the AgNP treatment.

After incubation of PBMCs with PVP-AgNPs and AgNO_3_ for 24 h, the total Ag content that was considered internalized or strongly bound to the cells (cell-associated) was measured using ICP-MS on digested samples. As shown in Figure 4, an increase in cellular association of Ag in PBMCs was observed with an increase in concentration from 10 µg·L^−1^ to 1000 µg·L^−1^ for both PVP-AgNPs and AgNO_3_ treatments, when compared to the negative control (*p* < 0.05). At concentrations of 500 and 1000 µg·L^−1^ there was a significant difference between the amount of Ag that was detected in the cells after Ag ion treatment and that after treatment with AgNPs (*p* < 0.05). No significant difference in the uptake of Ag in PBMCs was detected after treatments with AgNP and AgNO_3_ at lower concentrations (10 and 100 µg·L^−1^).

Ag content was measured in the supernatant after sample digestion using ICP-MS and a mass balance was calculated. Mass balance results (Table 2) indicate that after 24 h of exposure at a concentration of 1000 µg·L^−1^, the measured internalized Ag was 0.5% and 1% of the total Ag after treatments with PVP-AgNPs and AgNO_3_, respectively. Furthermore, at a concentration of 1000 µg·L^−1^, 42% and 16.6%, respectively, of the total Ag was detected in the supernatant of NPs and dissolved Ag. Supporting Information File 1, Figure S3 shows the Ag content that was internalized or attached to the cell surface of PBMCs after treatment with AgNPs and AgNO_3_ in each of six individuals. Mass balance data for each person after AgNP and AgNO_3_ treatments are presented in Table S1 (Supporting Information File 1). At concentrations of 10, 100, 500 and 1000 µg·L^−1^ PVP-AgNPs, the ratio between cellular uptake and content in the supernatant did not show any significant increase or trend. However, for the AgNO_3_ treatment, this ratio increased in a dose-dependent manner (Table 2).

**Table 2:** Mass balance of Ag. Ag mass balance (in the supernatant and cells) after AgNP and AgNO_3_ treatment measured by ICP-MS. Cell data were collected after three washes with PBS and shows internalized and strongly bound Ag. Total loss indicates losing some Ag during washes or experimental process. Ratio between uptake in cells and in supernatant in percent for Ag.

		Mass of Ag in exposure (ng)			
		
		10	100	500	1000

PVP-AgNP	cell	0.07	0.39	2.40	4.97
	supernatant	5.17	42.46	212.77	419.88
	total loss	4.79	57.15	284.83	575.15
	cell/Sup. (%)	1.32	0.91	1.13	1.18

AgNO_4_	cell	0.07	0.36	4.56	10.63
	supernatant	5.49	59.78	120.34	163.68
	total loss	4.44	39.87	375.10	825.69
	cell/Sup. (%)	1.18	0.61	3.79	6.49

### Impact of PVP-AgNPs and Ag ions on viability and metabolic activity of PBMCs

**Figure 5:**
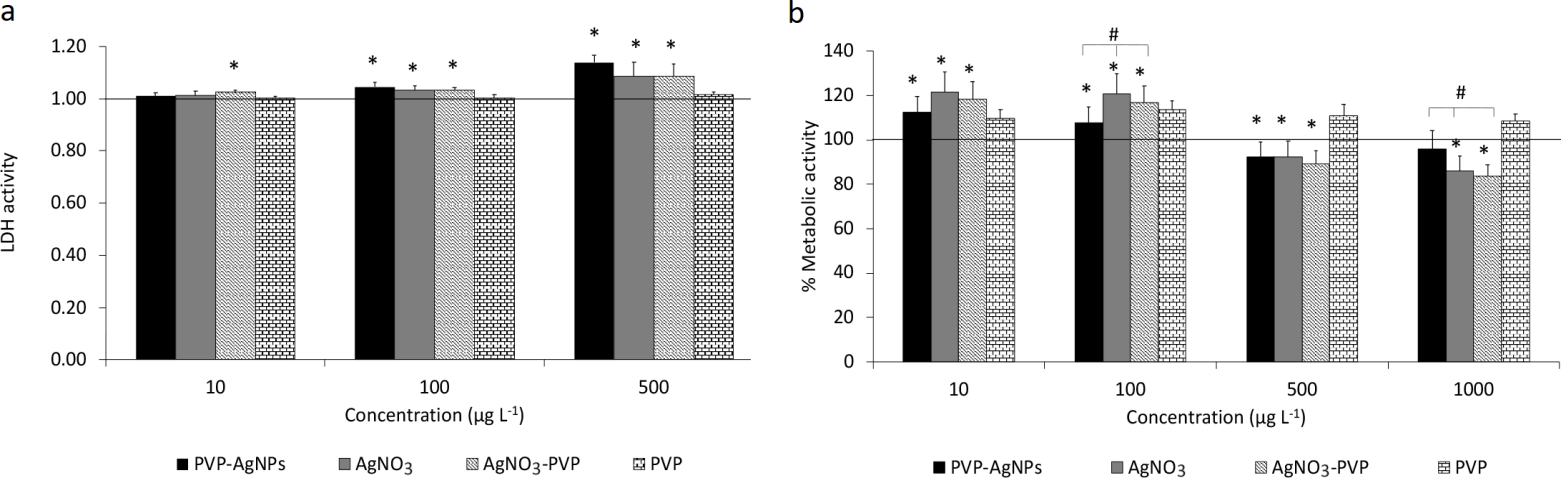
Effects of PVP-AgNPs, AgNO_3_, AgNO_3_-PVP, and PVP on PBMCs. PBMCs were exposed for 24 h to different concentrations of PVP-AgNPs, AgNO_3_, AgNO_3_-PVP, and PVP (0–1000 µg·L^−1^) in RPMI. The effect on cell viability was measured using LDH leakage (a). Cell metabolic activity was assessed by MTS assay (b) and the results are expressed as percentage of reduction compared to untreated cells. Data are presented as mean ± SE as the average of six independent experiments (*n* = 6). * shows a statistically significant difference from the control (0), and # shows a statistically significant difference when compared with PVP-AgNPs (*p* < 0.05).

Cell membrane integrity as a marker for cell viability was measured by LDH release; a greater LDH release is an indication of more damaged/dead cells. PBMCs were exposed to PVP-AgNPs, AgNO_3_, AgNO_3_-PVP and PVP at concentrations of 0 to 1000 µg·L^−1^. AgNO_3_ was used as a positive control and AgNO_3_-PVP was used to examine possible interactions between Ag ions and PVP in cell toxicity. In PBMCs exposed to PVP, no significant increase in LDH release was detected at any concentration. Both PVP-AgNPs and AgNO_3_ increased the LDH release in a dose-dependent manner compared to the control (without Ag), beginning from 100 µg·L^−1^ (Figure 5a). No significant toxicity was observed at 10 µg·L^−1^ concentration. The LDH test for AgNO_3_-PVP showed a significant toxicity effect at all concentrations. No significant difference was observed between treatments with PVP-AgNP and AgNO_3_ (or AgNO_3_-PVP) at any concentration.

To study the effect of Ag on PBMCs the metabolic activity was measured by the MTS assay after 24 h of exposure. PVP alone had no significant effect on the metabolic activity at any concentration. The metabolic activity of cells was elevated for all treatments at 10 and 100 µg·L^−1^ compared to the control (0) (Figure 5b). A significant decrease in cell metabolic activity after treatment with PVP-AgNPs, AgNO_3_, and AgNO_3_-PVP occurred at 500 and 1000 µg·L^−1^. At 1000 µg·L^−1^ Ag concentration, a significant difference was observed among the treatments with AgNO_3_, AgNO_3_-PVPs and PVP-AgNPs. The highest reduction in metabolic activity was found after treatment with AgNO_3_-PVPs, which elicited a 20% decrease in metabolic activity compared to the control group. Results for cell viability and metabolic activity of PBMCs for each person are presented in Supporting Information File 1, Figure S4 and Figure S5, respectively.

**Figure 6:**
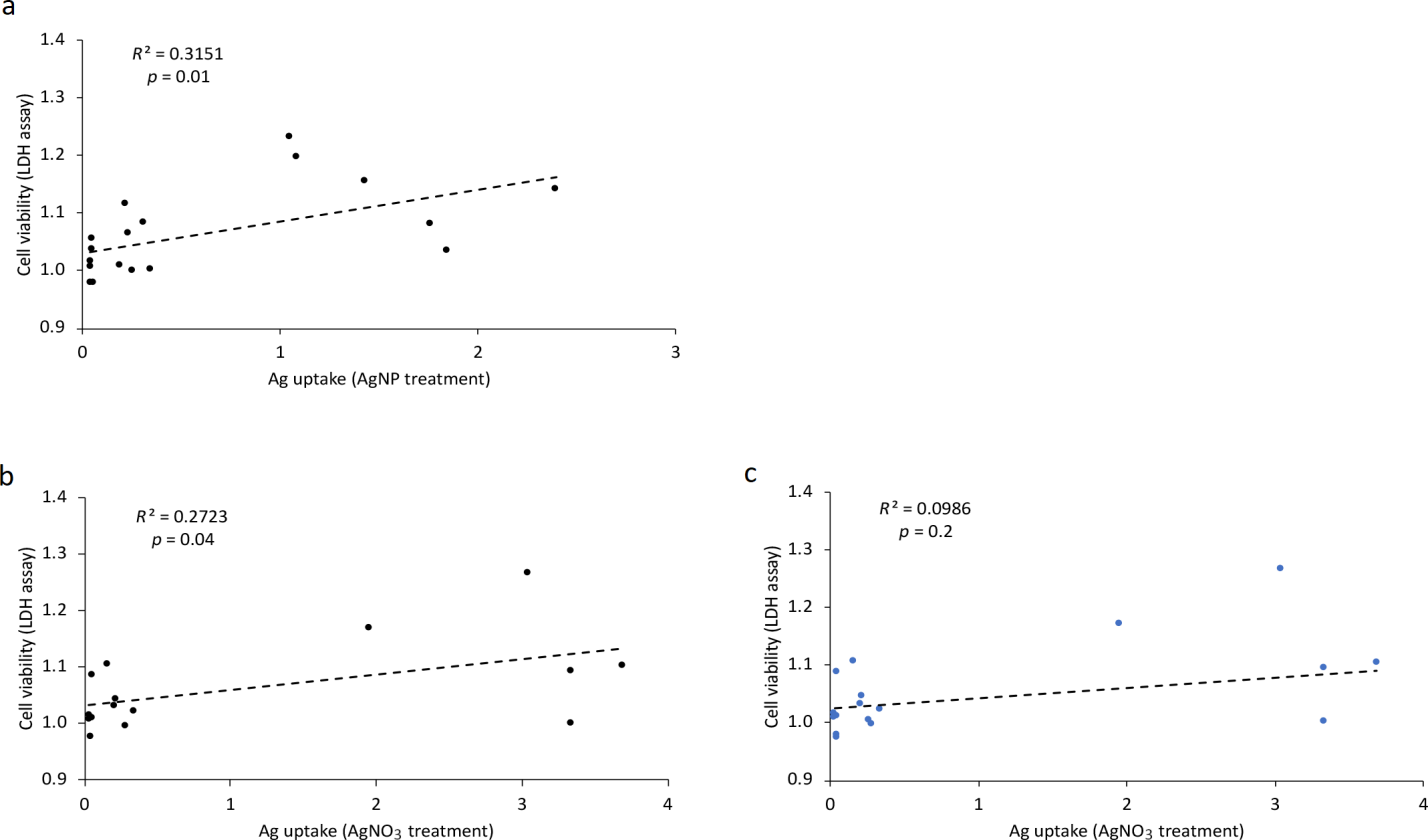
Pearson correlation between uptake and cytotoxicity (LDH assay) of Ag. Correlation between uptake and toxicity of Ag after treatment with AgNPs (a) and AgNO_3_ (b, c). There was a moderate positive correlation between the two variables (uptake and toxicity) for AgNPs: *r* = 0.56, *p* = 0.01 (a) and AgNO_3_ excluding Person 5: *r* = 0.52, *p* = 0.04 (b). No correlation was detected in AgNO_3_ including Person 5: *r* = 0.314, *p* = 0.2 (c).

The Pearson correlation coefficient was calculated to assess the relationship between uptake and cytotoxicity (LDH assay) of PVP-AgNPs and AgNO_3_ (Figure 6). A moderate but significant positive correlation was observed between uptake and toxicity of AgNPs (*r* = 0.56; *p* = 0.01). After AgNO_3_ treatment, Person 5 displayed a different trend in cell viability and Pearson correlation; considering Person 5 in the correlation, uptake and cytotoxicity did not show a significant relationship (*r* = 0.314; *p* = 0.2). However, excluding Person 5 from analysis changed the correlation to a moderate but significant correlation between uptake and toxicity (*r* = 0.52; *p* = 0.04). For comparison of the repeated measurements, we also calculated the *p* values of the random effects in order to determine if there is a significant variation across individuals. The range of all *p* values is between 0.0608 and 0.1496, indicating that there is no significant heterogeneity among the individuals, but that it was borderline significant in the former case.

## Discussion

Because of the antibacterial properties of AgNPs and their use in medical applications, there have been several studies on their effects on human blood cells [26–28]. Most of the previous studies investigating uptake and toxicity of AgNPs in human PBMCs have failed to report procurement/synthesis characterization, to examine the physicochemical transformations of NPs during the exposure time in the exposure medium, and/or to use physiologically relevant Ag concentrations. Therefore, the purpose of this study was to investigate the uptake and toxicity of well-characterized AgNPs in primary PBMCs from human volunteers at low and realistic concentrations.

Prior to investigating uptake and cytotoxicity of AgNPs to human cells, PVP-coated AgNPs were characterized using a multimethod approach. A previous detailed analysis reported that one of the reason for increase in size and polydispersity of NPs are related to Ostwald ripening of NPs [36], as evidenced in our STEM results. The subsequent reduction in size is explained as further transformations that are dominated by dissolution [36]. UV–vis spectroscopy data suggest some aggregation and possible shape changes after reprecipitation at higher concentrations, which could not be analyzed at lower concentrations due to limits to analytical sensitivity. This interpretation is supported by UV–vis spectra. Although not directly studied, dissolved silver chloride complexes and other particles were also likely formed [37], along with Ag binding to proteins present in the RPMI. Formation of dissolved silver chloride was suggested by speciation calculations (data not reported, as the accuracy of calculations can be questioned, given the media complexity and difference from those on which the model was validated). There is limited evidence that aggregation played a large role in NP transformations, except at high concentrations, due to protection by the PVP, and at later times because of possible protein interactions in the RPMI medium [38–39]. This result is in agreement with previous studies that found that aggregation/agglomeration of PVP-coated AgNPs was limited in biological media [40–44].

Dissolved oxygen in the solutions tend to oxidize AgNPs resulting in Ag ion release from the NP surface [45] and this was partially observed here. It has been suggested that the toxicity of Ag is linked to Ag ion release and availability [39,45–46]. As the exposure medium (RPMI) is a complex culture medium that contains different proteins and factors, alteration of the NP surface is likely leading to changes in the possible mechanisms responsible for uptake and toxicity. Ag release during the exposure time in RPMI medium was measured and little or no dissolved (below 3 kDa) Ag was detected after AgNP treatment, which suggests that minimal dissolution occurs. These results suggest that, although dissolution occurs, Ag ions are precipitated most likely as silver chloride (on the surface of the initial Ag particles, i.e., passivation, or as separate particles) or are bound to proteins. These leads to the formation of secondary NPs, chloro complexes and protein interactions in the RPMI medium yielding a Ag compounds that have a molecular mass above the nominal value of the 3 kDa filter membrane, which affects filter permeation. RPMI medium contains chloride, which can lead to reprecipitation of Ag ions as described previously [37]. Furthermore, cysteine and thiol-containing proteins are found in FBS and have a high affinity for Ag ions and NPs. Ag loss to the container wall could be another reason that we did not get full recovery of Ag, as it was discussed in [47]. This may lead to decreased Ag ion availability and possible NP surface interactions with the aqueous phase, ultimately reducing toxicity [10,48]. A Ag mass balance was calculated from measurement of filtrate and retentate. Results indicate that the PVP-coated AgNPs are more persistent in the exposure medium, whereas dissolved Ag is rapidly transformed. This was also reported by others authors [26,44].

Cell-associated Ag (operationally defined as internalized or strongly bound) in PBMCs after 24 h of exposure to PVP-AgNPs and dissolved Ag was measured using ICP-MS after washing. For both AgNP and Ag ion exposure, the amount of Ag that was taken up by cells increased as the Ag concentration increased, which is expected and consistent with previous literature [49–50]. There was a significant difference between Ag that was detected in the cells (or bound strongly to the cells) after AgNO_3_ treatment compared to NP treatment at concentrations of 500 and 1000 µg·L^−1^, which is consistent with findings from previous studies [28]. The higher cellular uptake of Ag in the form of ions is possibly related to a higher uptake rate for dissolved Ag [51] or because of the effect of cell type and the kinetics of cellular uptake or sorption [40]. Human PBMCs primarily consist of monocytes and lymphocytes (mostly T cells). Monocytes are known for their phagocytic properties and phagocytosis is suggested as one of the possible mechanisms for Ag uptake by cells [7]. As expected, it has been reported that in PBMCs exposed to AgNPs and AgNO_3_, monocytes ‘accumulate’ more Ag in the form of NPs than T lymphocytes [28,40]. However, in provided blood samples we did not have any information regarding the health status or diet of blood donors or the ratio of cell types. Thus, it is possible that the number of monocytes in the blood of different individuals could be elevated due to potential chronic inflammatory diseases or viral infection. The transformation of AgNPs to Ag ions may cause toxicity that is referred to as Trojan Horse mechanism, that is, AgNPs act as a carrier for Ag ions released after cell interactions [52–53]. Supporting Information File 1, Figure S3 shows different Ag uptake levels for each of six individuals.

In this study LDH toxicity assay was used for measuring cell viability and MTS assay for assessment of the cell metabolic activity. Metabolic activity of cells was stimulated for all treatments at 10 and 100 µg·L^−1^ compared to the control group. This agrees with previous results where an increase in cell activity at sub-lethal concentrations of AgNPs in hMSCs was reported by Greulich et al. [39]. This hormetic effect is a stimulatory effect by low concentrations of a potential toxin [48]. Cell viability assays of PBMCs exposed to AgNPs and Ag ions in the concentration range of 0–1000 µg·L^−1^ revealed a dose-dependent cytotoxicity during 24 h for both PVP-AgNPs and AgNO_3_, corroborating the results of Orta-García et al., who investigated the cytotoxicity effects of Ag nanoclusters (smaller than 2 nm) on PBMCs [25]. The concentrations of Ag tested in this study were much lower than the doses tested in previously published papers (up to 200 µg·L^−1^) [27,30,54]. LDH and MTS results are in agreement after exposure to PVP-AgNPs, AgNO_3_, and AgNO_3_-PVP at 500 µg·L^−1^, which means a decrease in the number of live cells leads to a decrease in metabolic activity. Interestingly, at a concentration of 100 µg·L^−1^, more damaged/dead cells were detected while the metabolic activity increased. The reasons are not clear but may be related to hormesis or to differential cellular uptake of NPs [55]. Artifacts in the LDH analysis were observed at the highest concentration and not reported.

In this study, we reported a cytotoxicity of AgNPs that started at low concentrations (100 µg·L^−1^). In some of the previous studies using the MTT assay for measuring cell viability, a decrease in cell viability (metabolic activity) was detected at very high concentrations of AgNPs [27,29]. There are several reasons that a decrease in MTT activity may not be seen even at high NP concentrations: 1) Using such a high NP concentration (1–15 mg·L^−1^) increases the possibility and rate of NP aggregation, resulting in less NPs available to the cells and therefore reducing the effective internalized concentration. 2) Cytotoxicity of Ag could increase mitochondrial activity in live cells but still lead to cell death. In this case, there might be a smaller number of live cells that is not reflected in mitochondrial activity. Therefore, prudent and careful interpretation of LDH and MTS (MTT) assays, along with other characterization data and biological endpoints should be considered.

In previous studies, it was shown that Ag ions are much more toxic than the same amount of Ag in the form of particulates [39,41,45]. In our study, using mass balance results, a cell-to-supernatant ratio was calculated for both AgNP and AgNO_3_ treatments. Results indicated a lower uptake of AgNPs compared with AgNO_3_ for the same added Ag mass. Comparing these results with cell viability measurements, which show the same cytotoxicity for both AgNP and AgNO_3_, suggested a higher toxicity of AgNPs for the same cell-associated mass of Ag. One possible mechanism is related to the Trojan Horse effect, with the NPs delivering a larger amount of ions per particle, overwhelming cell defenses.

The blood sample of Person 5 showed a different trend in the LDH assay than the other samples and it is not clear if this discrepancy was a result of variability between individuals. Excluding the sample of Person 5 from the calculations, the Pearson correlation coefficient indicated a moderate correlation between uptake and cytotoxicity of both AgNPs and AgNO_3_. Results from ANOVA with random effects for all data in cell toxicity (LDH) and cell metabolic activity (MTS) suggest that there may be an impact of individual variability on Ag toxicity and metabolic activity. A larger sample size is needed to fully identify or rule this out.

## Conclusion

Uptake and cytotoxicity of AgNPs in human PBMCs were investigated using well-characterized PVP-coated AgNPs along with AgNO_3_ controls. Our study showed that PVP-AgNPs do transform but are more stable in RPMI medium than Ag ions. In terms of overall observed toxicity, AgNPs and AgNO_3_ showed the same cytotoxicity. However, when the toxicity was normalized to the amount of cell-associated Ag, AgNPs were shown to be more toxic to PBMCs. Possible individual variability in terms of uptake and toxicity was indicated but would require a larger sample sizes for a more comprehensive test.

## Experimental

### Synthesis and characterization of PVP-AgNPs

Citrate-capped AgNPs (cit-AgNPs) were synthesized by the standard reduction of silver nitrate (AgNO_3_) in trisodium citrate as described in previous publications [56–58]. Briefly, separate solutions of AgNO_3_, trisodium citrate, and the reducing agent sodium borohydride (NaBH_4_) were prepared. AgNO_3_ and trisodium citrate were mixed together while stirring vigorously and NaBH_4_ was added in a dropwise fashion. The solution was heated slowly to boiling, boiled for a further 90 min, and then left overnight to cool in the dark. After synthesis, the NPs were washed by ultrafiltration (3 kDa cellulose membrane EMD Millipore) using a diafiltration technique to avoid drying and NP aggregation as well as to remove any residual reactants. The washing process was repeated at least three times and after each wash the trisodium citrate solution was added to make the original volume. A citrate solution was used to reduce NP growth/aggregation due to re-equilibration and loss of surface citrate [59]. The final reactant concentrations were ≪1% of the original. Cit-AgNPs were then recapped with PVP using the ligand exchange approach and DLS and UV–vis were used to quantify recapping, which was successful [42,56]. A solution of PVP10 (*M*_w_ = 10000, Sigma-Aldrich) was added to the NP batch while stirring vigorously for one hour. PVP is a non-toxic polymer [60–61] used to sterically stabilize particles by strongly binding to the AgNP core [56–57] and protect them from dissolution and aggregation in complex media [42–43].

*Z*-average hydrodynamic diameter and PDI of pristine PVP-AgNPs were measured using DLS with a Malvern Zetasizer NanoZS (Malvern Instruments, MA, USA). The colloidal stability (zeta potential) of PVP-AgNPs was measured by laser Doppler electrophoresis (Malvern Zetasizer NanoZS, MA, USA). The mean of at least three consecutive measurements was reported. DLS data for AgNPs in RPMI medium was not included because of the low concentrations used. Absorption spectra of PVP-AgNPs was recorded in triplicate over the wavelength range of 200–800 nm using a UV–vis spectrometer (UV-2600, Shimadzu Co., Kyoto, Japan). TEM and STEM samples were prepared using an ultracentrifugation-based method in UHPW and RPMI1640 medium (Sigma-Aldrich, Taufkirchen, Germany) supplemented with 10% fetal bovine serum (FBS; Gibco by Life Technologies) and 100 IU/mL pen/strep (Gibco by Life Technologies), respectively [62]. Briefly, a carbon-coated copper grid (300 mesh; Agar scientific) was placed into a clear plastic centrifuge tube and 4 mL of sample was added into the tube very slowly. The tube was covered by parafilm and ultracentrifuged for 1 h at 150,000*g* using a Beckman ultracentrifuge (L7-65 Ultracentrifuge). The grid was rinsed thoroughly and carefully with high-purity water and left overnight, covered to fully dry. The grids were then imaged by a Hitachi HT8700 and HD2000. The PVP-AgNP batch was checked for purity using energy-dispersive X-ray spectrometry. The final concentration of the PVP-AgNP batch was measured using inductively coupled plasma optical emission spectrometry (ICP-OES; Varian 710-ES). PVP-AgNP characterization in RPMI medium without cells was conducted using UV–vis spectrometer (extinction spectra of PVP-AgNPs aliquots collected at different time points following suspension in RPMI medium) and STEM. Ag concentration were measured at relevant exposure concentrations of PVP-AgNPs at *t* = 0 and at *t* = 24 h using ICP-MS (inductively coupled plasma mass spectrometry – NexION 350D, Perkin Elmer Inc., Massachusetts, USA).

### Measurement of the concentration of total Ag, dissolved Ag, and NP fraction

When exposed to oxygen, AgNPs oxidize through oxidative dissolution mechanisms and Ag ions are released from the surface of AgNPs to the solution [28,63]. To determine the extent of dissolved silver released from PVP-AgNPs in exposure medium (without cells), we used a centrifugal ultrafiltration method [35]. Aliquots of samples were taken at *t* = 0 and *t* = 24 h in water and medium and were subsequently added to the top of centrifugal ultrafiltration units (Pall Corporation, Microsep Advance with 3 kDa omega; maximum volume 4 mL) and centrifuged at 3250*g* for 15 min at 20 °C (Eppendorf 5810R centrifuge) [35,64]. The original (total Ag) and ultra-filtered (dissolved Ag) samples were then acidified with concentrated nitric acid (70% HNO_3_) and diluted to 1% acid prior to the measurement of Ag concentration with ICP-MS. Each sample was measured in triplicate and indium (^115^In) was used as an internal standard. The detection limit was calculated as 0.024 µg Ag·L^−1^. The validity of analytical method was checked every twelve measurements with two quality controls (Blank and 5 µg Ag·L^−1^). A serial dilution of Ag standard solution (1000 µg·mL^−1^, VWR analytical, USA) was used for the calibration curve.

To measure and compare the loss of Ag (dissolved or PVP-AgNPs) on the ultrafilter membrane or sorption to the exposure tube, 500 and 1000 µg·L^−1^ concentrations of Ag (as AgNO_3_) was prepared in UHPW and RPMI medium. Samples were collected at *t* = 0 and *t* = 24 h and were ultra-filtered and measured by ICP-MS in triplicate as previously described and were used to correct samples results. We corrected for any absorbed loss of Ag ion into the membrane.

### Isolation and culture of peripheral blood mononuclear cells (PBMCs)

Human blood from six anonymous individuals was purchased from the New York Blood Bank (New York City, NY). Since the blood samples from the “Blood Bank” are deidentified samples, this project is exempted from human subject research. PBMCs were isolated by gradient centrifugation using Ficoll-paque Plus (GE Healthcare Bio-Sciences AB, Sweden) [65–66]. In brief, peripheral blood from healthy volunteers was diluted with an equal volume of sterile phosphate-buffered saline (PBS; Gibco by life Technologies) and slowly layered onto the Ficoll-Paque media solution. Tubes were centrifuged at 500*g* for 30 to 40 min at 18 °C. After centrifugation a thin layer of mononuclear cells could be detected between plasma and Ficoll-Paque media. The layer of mononuclear cells was transferred to a sterile tube and was washed with sterile PBS three times. After the last wash, cells were resuspended in RPMI1640, supplemented with 10% fetal bovine serum and counted using a hemocytometer. This method led to more than 97% pure and live cells. Freshly isolated PBMCs were adjusted to 2.5 × 10^5^ in 24 well-plates (Costar, ME, USA) for cell culture and bio-uptake experiments, and 2.5 × 10^4^ cells in 96 well-plates (Corning Incorporated, NY, USA) for cell viability and metabolic activity test.

### Uptake of Ag into PBMCs and mass balance

ICP-MS was used to quantify the uptake of Ag in PBMCs after exposure to PVP-AgNPs and AgNO_3_ (as control). Freshly isolated PBMCs were resuspended in RPMI medium and then seeded in 24-well cell culture plates at a density of 2.5 × 10^5^ cells. PVP-AgNP and AgNO_3_ stock (25 mg·L^−1^) were diluted with cell-culture water (Sigma-Aldrich, Taufkirchen, Germany) and added to attain final concentrations of 10, 100, 500, and 1000 µg·L^−1^ prior to exposure. The AgNP solution was sonicated for 20 min to reduce NP agglomeration immediately before exposure. The plates were then incubated for 24 h at 37 °C and 5% CO_2_. Each exposure study was run in triplicate. After 24 h each well was collected, and the cells and supernatant were separated using a centrifuge (300*g*, 10 min). The supernatant was used for Ag analysis to determine the amount of Ag that was not taken up by PBMCs. Cells were washed three times with PBS using a centrifuge (300*g*, 15 min) and then lysed and digested with concentrated HNO_3_. The Ag ion or AgNP uptake by cells was measured by quantifying the total Ag concentration in the digested samples using ICP-MS.

### Measurements of viability and metabolic activity of cells

To investigate the cytotoxicity of Ag on PBMCs we used two different assays that measure cell damage (LDH assay) and metabolic activity (MTS assay). LDH is a colorimetric assay that measures the membrane integrity and quantifies lactate as a substrate that is released from lysed cells into the medium. LDH cytotoxicity assay (Dojindo laboratories, Kyoto, Japan) was performed according to the manufacturer’s instructions. In brief, immediately after isolation of PBMCs, cells were treated in the absence (control) and presence of increasing doses of PVP-AgNPs, AgNO_3_ (as positive control), AgNO_3_-PVP and PVP (10, 100, 500, and 1000 µg·L^−1^) in 96-well plates for 24 h in six replicates. Subsequently, 100 µL of the assay buffer was added to each well and incubated at room temperature in the dark. After 30 min, 50 µL of the stop solution was added to each well and the LDH activity of the samples was assessed by measuring NADH absorbance at 490 nm.

Cell metabolic activity was measured using 3-(4,5-dimethylthiazol-2-yl)-5-(3-carboxymethoxyphenyl)-2-(4-sulfophenyl)-2*H*-tetrazolium (MTS; Promega, Madison, WI) assay. The MTS assay measures the insoluble formazan that is accumulated in the metabolically active cells. The MTS assay was performed according to the manufacturer’s protocol. Briefly, isolated PBMCs were exposed to 0, 10, 100, 500, and 1000 µg·L^−1^ of PVP-AgNPs, AgNO_3_, AgNO_3_-PVP and PVP in 96-well plates in six replicates for 24 h. Subsequently, MTS dye was added to each well, and incubated at 37 °C in a 5% CO_2_ humidified incubator for four additional hours. The optical density of reduced MTS was measured at 490 nm using a 96-well plate reader spectrophotometer. The potential interference of particles with LDH and MTS assay was examined in cell-free conditions and correction was applied where necessary.

### Statistical analysis

Data for uptake, cell viability and metabolic activity are expressed as mean ± standard error of mean (SEM) for six separate donors. As there were multiple measurements for each donor, the potential correlations among those measurements should be considered. To accommodate the correlations of measurements within each subject and the variations across different subjects, we utilized the ANOVA with random effects for multiple comparisons to detect the significant differences between the treatment and control groups, and between different concentration groups within a treatment. The Pearson’s correlation was calculated to measure the relationship between two variables. All values of *p* below 0.05 were considered statistically significant. All analyzes were performed using SAS software (SAS 9.4).

## Supporting Information

File 1 Supplementary figures and tables.
