# Supplementary material for: Characterization, bio-uptake and toxicity of polymer-coated silver nanoparticles and their interaction with human peripheral blood mononuclear cells
Source: Beilstein J Nanotechnol. 2021 Mar 24;12:282–94. doi: 10.3762/bjnano.12.23 (PMC8008093; doi:10.3762/bjnano.12.23)
Supplement: File 1 — Supplementary figures and tables. [file Beilstein_J_Nanotechnol-12-282-s001.pdf]

BEILSTEIN JOURNAL OF NANOTECHNOLOGY

Supporting Information

for

Characterization, bio-uptake and toxicity of polymer-coated silver nanoparticles and their interaction with human peripheral blood mononuclear cells

Sahar Pourhoseini, Reilly T. Enos, Angela E. Murphy, Bo Cai and Jamie R. Lead

Beilstein J. Nanotechnol. **2021,** *12,* 282–294. doi:10.3762/bjnano.12.23

Supplementary figures and tables

License and Terms: This is a supporting information file under the terms of the Creative Commons Attribution License (https://creativecommons.org/licenses/by/4.0). Please note that the reuse, redistribution and reproduction in particular requires that the author(s) and source are credited and that individual graphics may be subject to special legal provisions.
The license is subject to the *Beilstein Journal of Nanotechnology* terms and conditions: (https://www.beilstein-journals.org/bjnano/terms)

Table S1: Extinction coefficients. Extinction coefficient calculated based on the Beer–Lambert law.

		Extinction Coefficients ($Lmg^{-1}cm^{-1}$)					
		0h	1h	3h	6h	12h	24h
Concentration ($\mu g\ L^{-1}$)	100 $\mu g\ L^{-1}$	8.88×10^{-3}	7.42×10^{-3}	8.97×10^{-3}	4.51×10^{-3}	6.59×10^{-3}	6.06×10^{-3}
	500 $\mu g\ L^{-1}$	2.47×10^{-3}	1.92×10^{-2}	1.31×10^{-3}	1.88×10^{-2}	2.12×10^{-3}	1.7×10^{-3}
	1000 $\mu g\ L^{-1}$	5.88×10^{-4}	1.04×10^{-3}	6.42×10^{-4}	7.36×10^{-4}	5.24×10^{-4}	9.23×10^{-4}

Table S2: Mass balance of Ag. Ag mass balance (ng) in 2.5×10^5 cells (absorbed or cell surface-attached) in AgNP and $AgNO_3$ treatments for each six individual measured by ICP-MS. Data are presented as mean ± standard error (*n* = 3).

Person 1		Mass of Ag in exposure (ng)			
		10	100	500	1000
PVP-AgNP	cell	0.06	0.31	1.76	5.41
	Supernatant	6.23	38.02	153.61	312.95
	Total loss	3.71	61.67	344.62	681.63
$AgNO_3$	cell	0.05	0.32	4.54	8.14
	Supernatant	5.85	57.03	120	119.42
	Total loss	4.1	42.65	375.46	872.43

Person 2		Mass of Ag in exposure (ng)			
		10	100	500	1000
PVP-AgNP	cell	0.06	0.34	2.13	4.57
	Supernatant	3.59	33.17	163.68	425.14
	Total loss	6.53	66.49	334.19	570.3
$AgNO_3$	cell	0.05	0.3	4.98	14.44
	Supernatant	6.21	41.29	119.86	142.15
	Total loss	3.74	58.4	375.16	843.42

Person 3		Mass of Ag in exposure (ng)			
		10	100	500	1000
PVP-AgNP	cell	0.07	0.51	2.75	5.96
	Supernatant	4.53	39.64	301.33	457.06
	Total loss	5.4	59.85	195.93	536.97
$AgNO_3$	cell	0.06	0.42	4.97	6.95
	Supernatant	4.78	43.79	121.87	198.16
	Total loss	5.16	55.79	373.16	794.88

Person 4		Mass of Ag in exposure (ng)			
		10	100	500	1000
PVP-AgNP	cell	0.07	0.46	3.58	6.78
	Supernatant	4.55	48.19	206.93	404.74
	Total loss	5.38	51.35	289.49	588.48
$AgNO_3$	cell	0.08	0.51	5.5	11.86
	Supernatant	5.35	70.86	119.85	163.53
	Total loss	4.57	28.64	374.65	824.61

Person 5		Mass of Ag in exposure (ng)			
		10	100	500	1000
PVP-AgNP	cell	0.08	0.38	2.63	4.31
	Supernatant	6.15	45.34	213.65	409.12
	Total loss	3.76	54.28	283.73	586.57
$AgNO_3$	cell	0.08	0.39	4.47	13.54
	Supernatant	5.19	69.46	120.05	158.99
	Total loss	4.73	30.16	375.48	827.47

Person 6		Mass of Ag in exposure (ng)			
		10	100	500	1000
PVP-AgNP	cell	0.07	0.32	1.56	2.79
	Supernatant	5.99	50.41	237.4	510.26
	Total loss	3.94	49.27	261.04	486.95
$AgNO_3$	cell	0.07	0.23	2.91	8.84
	Supernatant	5.57	76.22	120.41	199.83
	Total loss	4.36	23.55	376.68	791.32

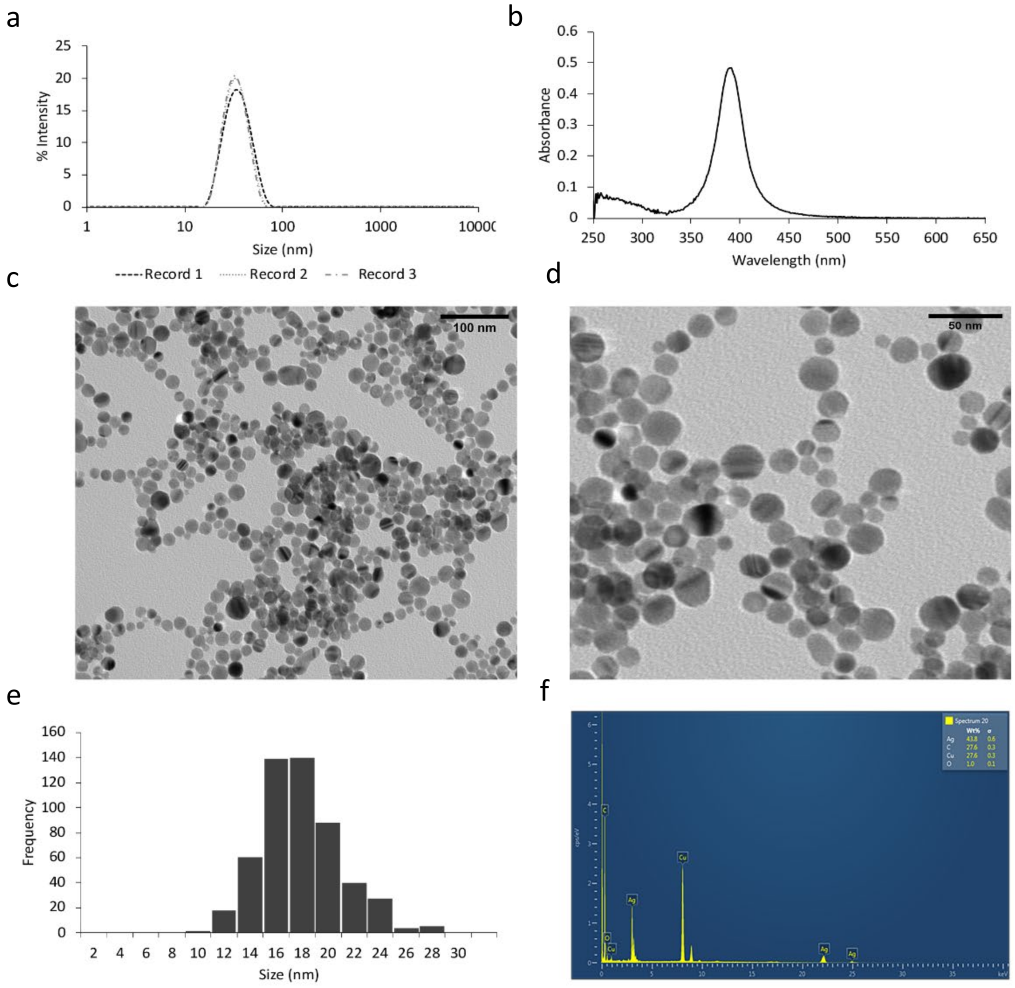

Figure S1: NP characterization results. Characterization of stock PVP-AgNPs using DLS (a), UV–vis spectra (b), TEM images with different magnifications (16.9 nm) and particle size distribution (c–e), and EDX imaging (f).

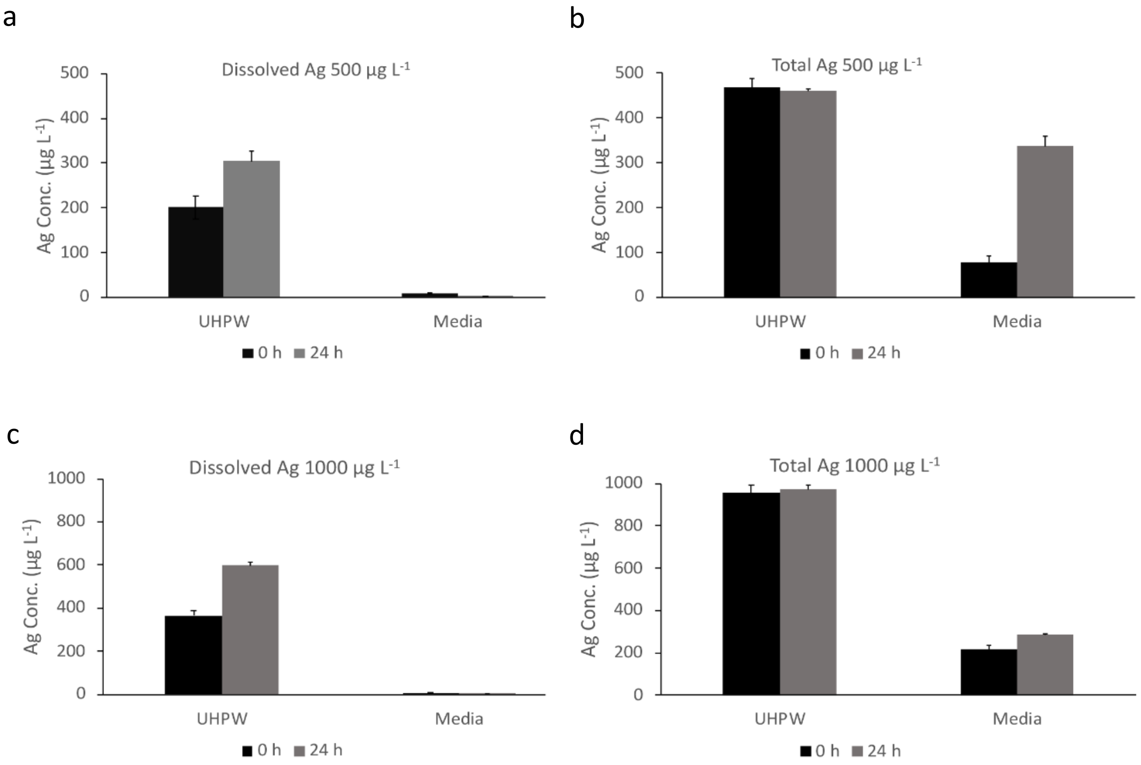

Figure S2: Dissolved and total concentration of Ag in $AgNO_3$ in UHPW and RPMI medium. Dissolved Ag concentration measurements using centrifugal ultrafiltration units (a and c) and total Ag concentration (b and d) in UHPW and RPMI medium at T = 0 and T = 24 h measured by ICP-MS. Data are reported as mean ± SE (n = 3).

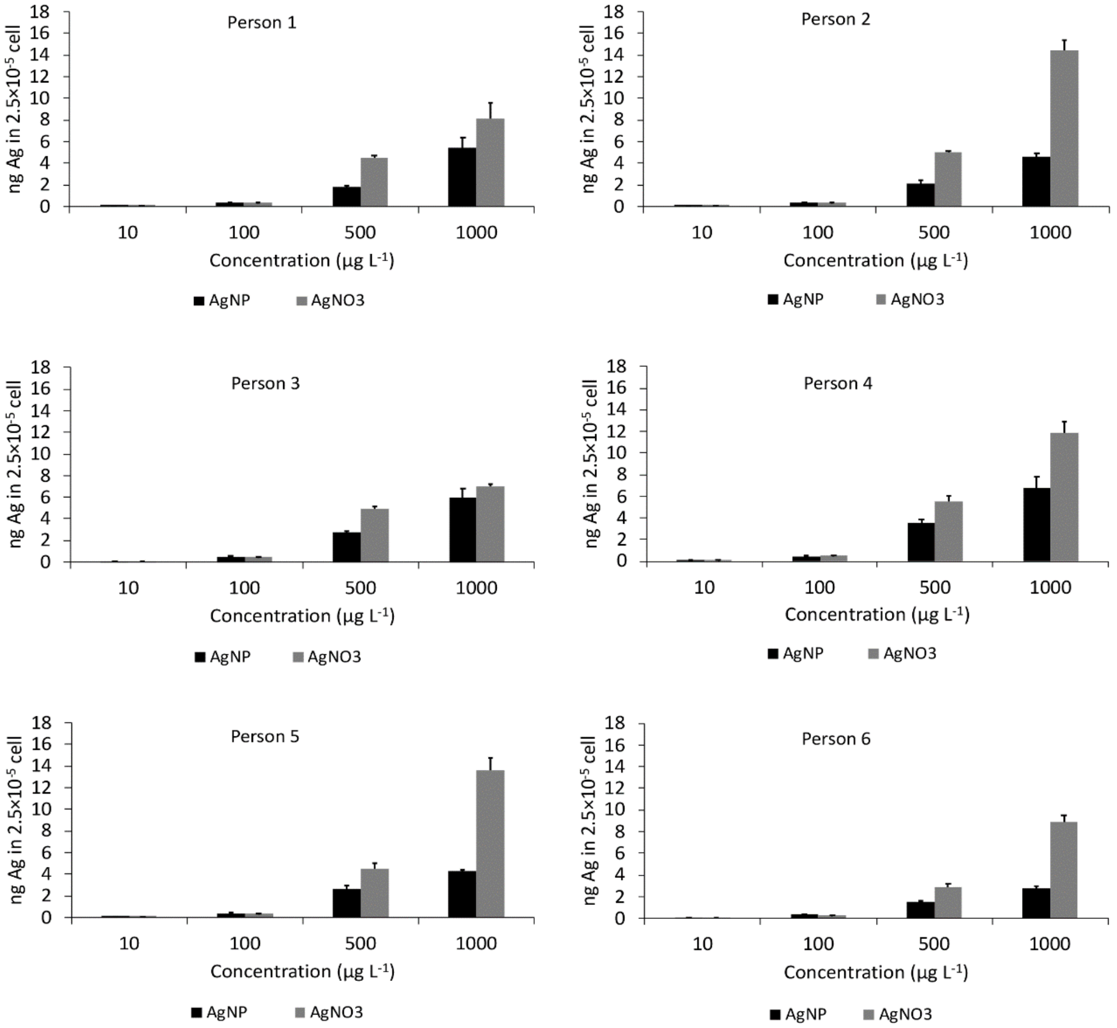

Figure S3: Ag uptake (ng) in 2.5×10^5 cells. Results for absorbed or cell surface-attached Ag after treatment of PBMCs in six individuals. The amount of Ag in cells after three washes with buffer was measured by ICP-MS.

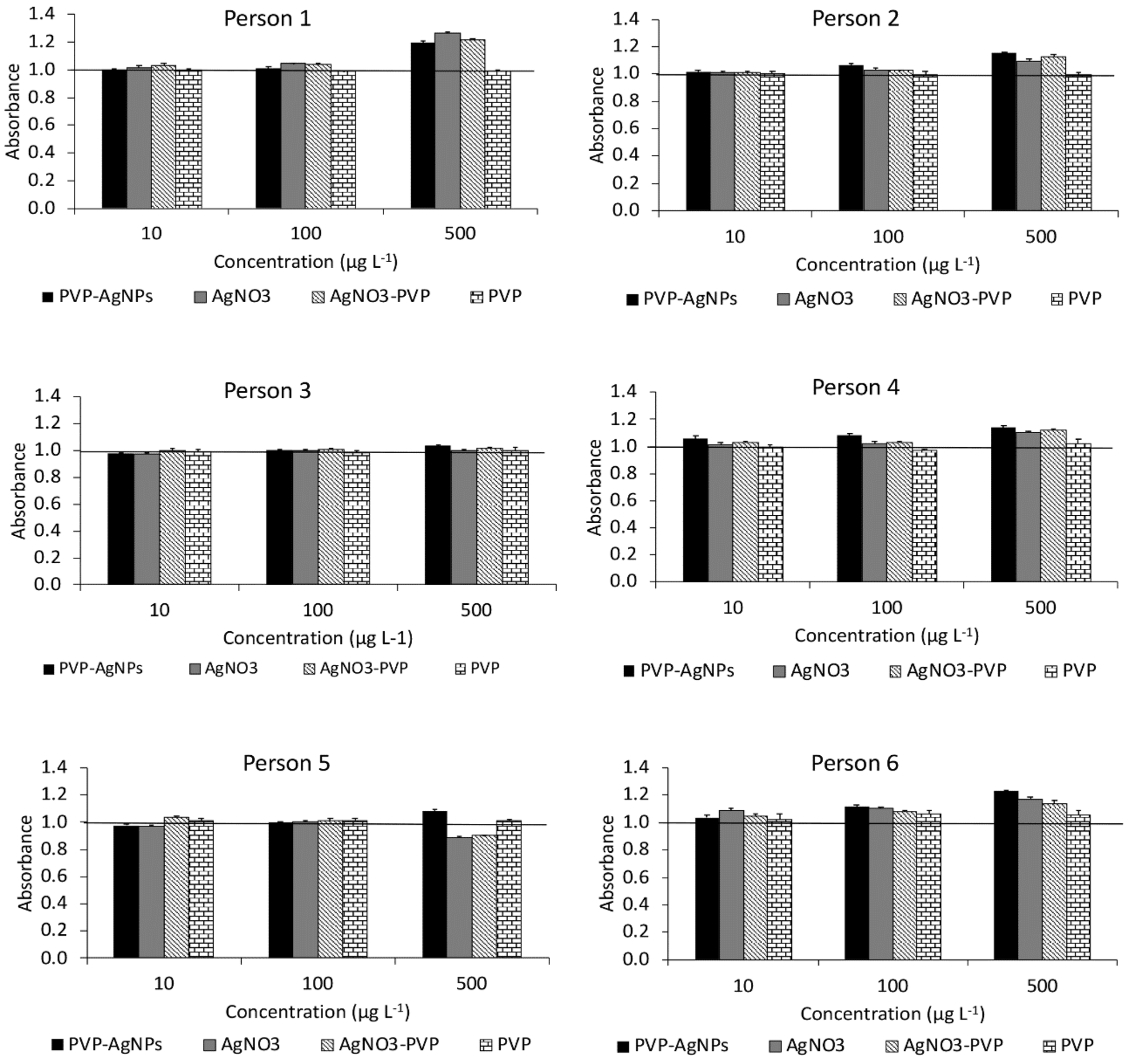

Figure S4: Cell viability test. Cell viability of PBMCs assessed by LDH leakage after 24 h of treatment with the given concentrations PVP-AgNPs, $AgNO_3$, $AgNO_3$-PVP, and PVP. Data represents the means of six replicates ± standard error.

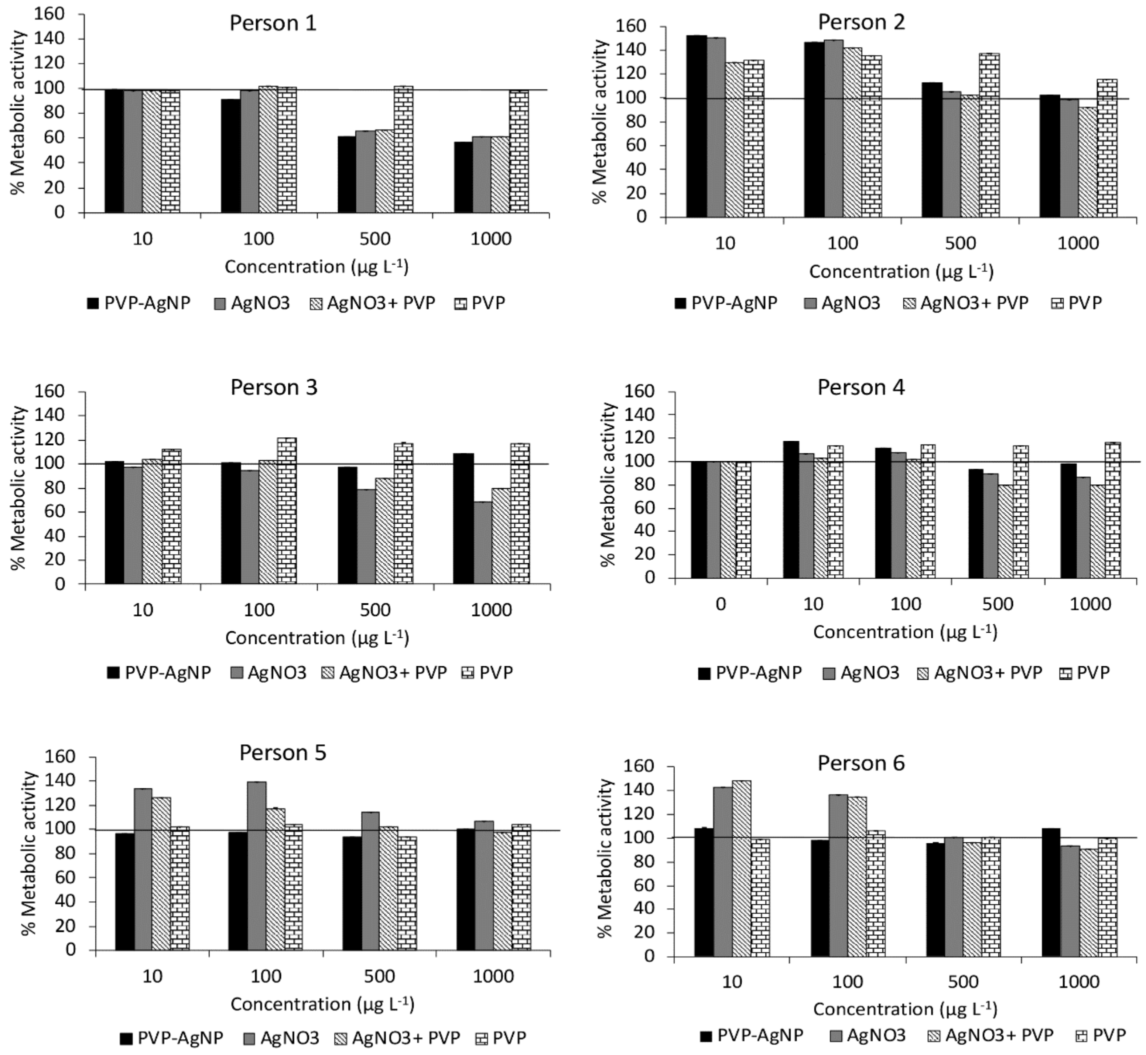

Figure S5: Cell metabolic activity. Metabolic activity of PBMCs measured by MTS assay after 24 h of exposure at the given concentrations for six individuals. Data represents the means of six replicates ± standard error.
